# Investigating a New Way to Assess Metabolic Risk in Pregnant Females with Prior RYGB Surgery

**DOI:** 10.3390/nu16162704

**Published:** 2024-08-14

**Authors:** Teresa Gisinger, Birgit Reiter, Karin Preindl, Thomas Stimpfl, Liliana-Imi Gard, Sabina Baumgartner-Parzer, Alexandra Kautzky-Willer, Michael Leutner

**Affiliations:** 1 Department of Internal Medicine III, Clinical Division of Endocrinology and Metabolism, Medical University of Vienna, 1090 Vienna, Austria; teresa.gisinger@meduniwien.ac.at (T.G.); liliana-imi.gard@meduniwien.ac.at (L.-I.G.); alexandra.kautzky-willer@meduniwien.ac.at (A.K.-W.); 2 Department of Laboratory Medicine, Medical University of Vienna, 1090 Vienna, Austria; birgit.reiter@meduniwien.ac.at (B.R.); karin.preindl@meduniwien.ac.at (K.P.); thomas.stimpfl@meduniwien.ac.at (T.S.); 3 Joint Metabolome Facility, Medical University of Vienna, 1090 Vienna, Austria

**Keywords:** metabolic risk, glucose metabolism, pregnancy, bariatric surgery

## Abstract

Background: Obesity in pregnancy is linked to adverse clinical outcomes such as gestational diabetes. Recently, a risk score calculated by different ceramide concentrations was recognized as a new way to investigate cardiovascular risk. The aim was to analyze if the ceramide risk score and cardiometabolic risk vary between normal-weight, obese, and females with prior Roux-en-Y bypass surgery (RYGB) during pregnancy. Methods: Three cohorts were investigated: first, 25 pregnant females with a history of RYGB; second, 19 with preconception BMI ≥ 35 kg/m^2^; and third, 19 normal-weight (preconception BMI < 25 kg/m^2^). Around the 24th to 28th weeks of gestation routine laboratory assessments, 3 h 75 g oral and intravenous glucose tolerance tests were carried out. The correlation of ceramide risk scores and ceramide ratios (Cer(d18:1/18:0)/Cer(d18:1/16:0)) with metabolic parameters was analyzed via Pearson correlation. The cohorts were compared via ANOVA and unpaired *t*-tests. Results: The RYGB cohort had lower ceramide risk scores and ratios compared to obese pregnant females (7.42 vs. 9.34, *p* = 0.025; 0.33 vs. 0.47, *p* < 0.001). Ceramide risk score and ratio were found to correlate negatively with insulin sensitivity (measured with the Matsuda (r = −0.376, *p* = 0.031; r = −0.455, *p* = 0.008) and calculated sensitivity index (r = −0.358, *p* = 0.044; r = −0.621, *p* < 0.001) in females without RYGB. The ceramide risk score correlated positively with body fat in RYGB females (r = 0.650, *p* = 0.012). Conclusions: We found that females after RYGB have lower ceramide risk scores and ceramide ratios compared to obese pregnant females, possibly indicating lower metabolic risk.

## 1. Introduction

The rate of obesity is constantly rising worldwide, especially in the US, where 42.4% of adults are obese [1]. This development might be explained by the increasing sedentary lifestyle and diets rich in calories [2]. Still, genetic factors and undernutrition in early life can also lead to obesity [3]. Obesity is known to be related to a higher risk of comorbidities, including type 2 diabetes mellitus, hypertension, coronary heart disease, cardiomyopathy, and heart failure [2]. Moreover, obesity is a risk factor for infertility, pregnancy loss, pregnancy complications, labor complications, and fetal and maternal death during pregnancy [4].

Nowadays, a commonly used treatment option against obesity is bariatric surgery, including Roux-en-Y bypass (RYGB) surgery. Bariatric surgery not only reduces body weight but also has positive effects on cardiovascular and metabolic parameters [5]. More specifically, previous studies investigated a reduced 10-year and lifetime cardiovascular disease risk after RYGB compared to individuals without bariatric surgery [5].

Concerning individuals with RYGB in pregnancy, a higher incidence of dumping syndrome and hypoglycemic events can be found [6,7]. This finding could be caused by an increased postprandial expression of glucagon-like peptide-1, followed by significant insulin secretion and therefore hypoglycemic events [8,9]. However, pregnant women after RYGB had lower risk of gestational diabetes and better insulin sensitivity compared to control subjects [10,11]. Moreover, lower levels in cholesterol, low-density lipoprotein (LDL) cholesterol, and triglycerides in pregnant females after RYGB compared to obese pregnant females were reported [12]. Further bariatric surgery is related to a lower risk of cardiovascular diseases, but the exact pathomechanism is still unclear [13].

Recently, new predictor values for cardiovascular risk were investigated, namely, ceramides. Ceramides consist of a sphingoid base and a fatty acyl chain. Depending on the fatty acyl chain, different subtypes are defined, and differences in cardiometabolic disease risk can be seen [14]. The Mayo Clinic already established, during a clinical routine, thresholds for ceramide risk scores, including ceramides with long fatty acyl chains to allocate individuals into different cardiovascular risk cohorts [15]. It is suggested that ceramides might also be a valid secondary predictive marker such as LDL cholesterol concerning cardiovascular risk [16,17]. Ceramides with long fatty acyl chains, particularly Cer(d18:1/16:0) and Cer(d18:1/18:0), are related to a higher risk of type 2 diabetes mellitus, fatty liver disease, hypertension, atherosclerosis, and heart failure [18,19]. In mouse and rat models, better insulin sensitivity, glucose tolerance, and diabetes mellitus outcome, less atherosclerotic plaque formation, and better hypertension and heart failure outcome were determined in Des1 knock-out animals, which have an inhibited ceramide synthesis [20,21,22]. Previous studies could find higher values of ceramides with long fatty acyl chains, namely, Cer(d18:1/18:0), Cer(d18:1/20:0), and Cer(d18:1/24:1), in patients with obesity and type 2 diabetes mellitus [23]. Further high values of Cer(d18:1/18:0), Cer(d18:1/20:0), and Cer(d18:1/24:1) were correlated with insulin resistance and inflammation [23]. The ratio of two ceramides (Cer(d18:1/18.0)/Cer(d18:1/16.0)) might be a predictor of type 2 diabetes mellitus when measuring ceramide concentrations via a high-throughput mass spectrometry approach [24]. Four ceramide concentrations—(Cer(d18:1/16:0), Cer(d18:1/18:0), Cer(d18:1/24:1), Cer(d18:0/24:0))—were also measured via liquid chromatography–tandem mass spectrometry assay in pregnant females [25]. These ceramides had higher levels in pregnancy compared to non-pregnant females, but still-higher concentrations were also related to gestational diabetes [25]. After gastric bypass, a reduction in insulin resistance associated with lower levels of plasma and urinary ceramides was reported [26,27]. In particular, it is postulated that ceramides inhibit the action of the anabolic enzyme Akt or protein kinase B, which is an insulin-stimulated kinase that acts in the liver by reducing gluconeogenesis, increases the glucose uptake in muscle and adipose tissue, and further represses the production of glucagon in pancreatic alpha-cells [18,28,29]. Moreover, past literature investigated that due to the pathological expansion of adipose tissue in metabolic syndrome or type 2 diabetes mellitus, numerous molecular actions of lipid storage, adipocyte turn-over, and hormone secretion are disturbed [30]. Therefore, uncommon lipid metabolism, leading to a specific lipid profile including ceramides, especially Cer(d18:1/16:0) and Cer(d18:1/18:0), takes place [30].

Data on ceramides in pregnant women with a history of RYGB surgery are not currently available. Hence, in the present study, we were interested in whether pregnant females with prior RYGB have differences in ceramide concentrations, including Cer(d18:1/16:0), Cer(d18:1/18:0), Cer(d18:1/24:1), and Cer(d18:1/24:0), as well as the above-described ceramide risk score and ceramide ratio (Cer(d18:1/18:0)/Cer(d18:1/16:0)), and therefore a different metabolic risk than obese pregnant females.

## 2. Materials and Methods

### 2.1. Study Participants

This study was carried out from April 2014 to February 2016 at the Division of Endocrinology and Metabolism, Medical University of Vienna. The detailed protocol and characteristics of study participants were already published [7,9,12]. In summary, in this study, 25 pregnant females with prior RYGB were included. Further, this study included 19 pregnant females with a pre-pregnancy BMI ≥ 35 kg/m^2^ and 19 normal-weight (preconception BMI < 25 kg/m^2^) pregnant females. The study visit took place between the 24th and 28th weeks of gestation. Females with infectious diseases, including hepatitis B or C, human immunodeficiency virus (HIV), or any liver or renal disease, were excluded. Also, an exclusion criterion for the RYGB cohort was any surgical procedures other than laparoscopic RYGB surgery. Further, females with prior insulin treatment were excluded. This study was approved by the Ethics Committee of the Medical University of Vienna (1364/2022, approved 21 July 2022) and was performed in accordance with the Declaration of Helsinki. Written informed consent to participate in this study was obtained from all participants.

At the study visits’ participants had routine laboratory assessments and anthropometric measurements. Moreover, 3 h 75 g oral and intravenous glucose tolerance tests were conducted. For the intravenous glucose tolerance test (IVGGT), a bolus of glucose standardized to the body size was injected intravenously, and glucose, insulin, and C-peptide concentrations were measured. During these tests, various laboratory measurements were taken, including gastric inhibitory polypeptide (GIP) and glucagon-like peptide 1 (GLP1). 

### 2.2. Laboratory Methods

Laboratory measurements, which are in line with the international standard laboratory methods at our certified Department of Medical and Chemical Laboratory Diagnostics (http://www.kimcl.at/, accessed on 12 August 2024), were taken at the study visit between the 24th and 28th week of gestation. GIP and GLP-1 were measured using the Human GIP (total) and GLP-1 (Active 7–36) ELISAs purchased from Millipore (Darmstadt, Germany) and from ALPCO Diagnostics (Salem, NH, USA), respectively. Insulin sensitivity was measured via previously described indices, namely, Matsuda, sensitivity index (SI), Pacini, and calculated sensitivity (CSI) indices. Therefore, the laboratory values from the IVGTT were used [31]. As previously described, the CSI is a solid alternative of clamp insulin sensitivity [31]. The disposition index was calculated as the product of CSI (×10^−4^ min^−1^ [pmol/L]^−1^) and AIR*g* (pmol/L) and indicates beta cell function in insulin resistance. 

Moreover, the HOMA-IR index was calculated to investigate hepatic insulin resistance. Based on the method by Kauhanen et al. [32], a liquid chromatography–tandem mass spectrometry process for the determination of N-palmitoyl-sphingosine (Cer(d18:1/16:0), N-stearoyl-sphingosine (Cer(d18:1/18:0), N-nervonoyl-sphingosine (Cer(d18:1/24:1), and N-lignoceroyl-sphingosine (Cer(d18:1/24:0) in EDTA plasma was developed and validated (detailed method parameters and validation data can be found in Supplementary Materials).

The concentrations of the four ceramides were used to calculate the ceramide risk score based on the MI heart ceramides index by the Mayo Clinic [33]. As previously described, the score was calculated by adding one point for each Cer(d18:1/16:0), Cer(d18:1/18:0), Cer(d18:1/24:0), or Cer(d18:1/24:1) value above the median and an additional point for each value in the fourth quartile [33]. Moreover, one point was added if each ratio value—Cer(d18:1/16:0)/Cer(d18:1/24:0), Cer(d18:1/18:0)/Cer(d18:1/24:0), or Cer(d18:1/24:1)/Cer(d18:1/24:0)—was in the third quartile, and two points were added for ratio values in the fourth quartile [33]. All these points were summed up to create the ceramide risk score. Moreover, the ratio of two ceramide concentrations (Cer(d18:1/18:0)/Cer(d18:1/16:0) was calculated as this ratio has previously been described to be correlated with a higher risk for diabetes mellitus [24].

### 2.3. Statistical Analysis

For descriptive analyses, continuous variables were reported by mean and standard deviation. The lipid and glucose parameters were compared via analysis of variance (ANOVA) between the different cohorts. All the lipid and glucose parameters were compared via unpaired *t*-tests between the RYGB, obese, and control cohort, and a Bonferroni-Holm correction was carried out. Further, the correlation between ceramide risk score and lipids, as well as glucose metabolism, was investigated via Pearson correlation in the RYBG cohort. Next, the correlation between the ceramide ratio and lipids and the glucose metabolism was also investigated via Pearson correlation in the RYGB cohort. Lastly, the same correlation analyses were carried out, combining both cohorts without bariatric surgery. In order to correlate the gastric inhibitory polypeptide, glucagon-like peptide, and glucagon values over a period of 120 min with the ceramide risk score and ratios, the area under the curve for each laboratory value was calculated. Analyses were carried out with R-Statistics Version 2024.04. *p*-values under 0.05 indicate statistical significance. 

## 3. Results

### 3.1. Baseline Characteristics

**Figure 1:**
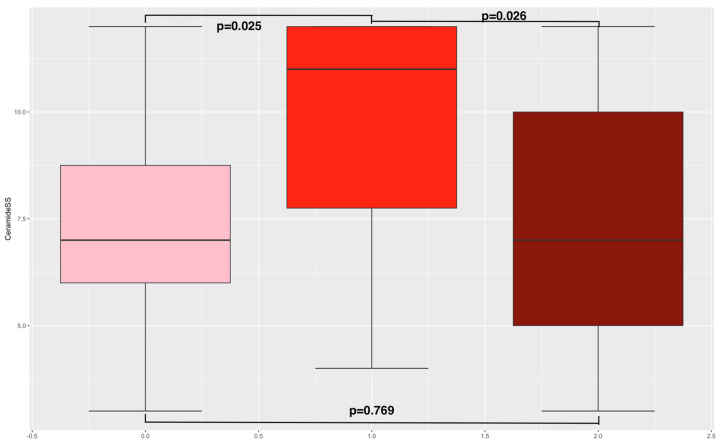
Ceramide risk score. This figure presents boxplots including the ceramide risk score distribution in the three study cohorts. The left boxplot indicates the RYGB cohort, the middle the obese, and the right the normal-weight cohort. The *p*-values for the unpaired *t*-test are also reported.

The mean age of the RYGB cohort was 31.9 years, and the mean BMI before pregnancy was 28.4 kg/m^2^. For the obese cohort, the mean age was 33.7 years, and the mean BMI before pregnancy 37.9 kg/m^2^. Lastly, the normal-weight cohort had a mean age of 29.7 years and a mean BMI of 21.7 kg/m^2^ before pregnancy (see Supplementary Table S6). As to the comparison of metabolic parameters between the pregnant females after RYGB, the obese pregnant females, and normal-weight pregnant females via ANOVA, the results have already been published [12]. In summary, no differences in age between the three groups were present. Compared to the normal-weight cohort, pregnant females after RYGB had lower levels of total and LDL cholesterol [12]. Within these groups, we found differences in ceramide risk score (obese cohort = 9.34; RYGB cohort = 7.42; control = 7.17, *p* = 0.033 (see Figure 1)).

**Figure 2:**
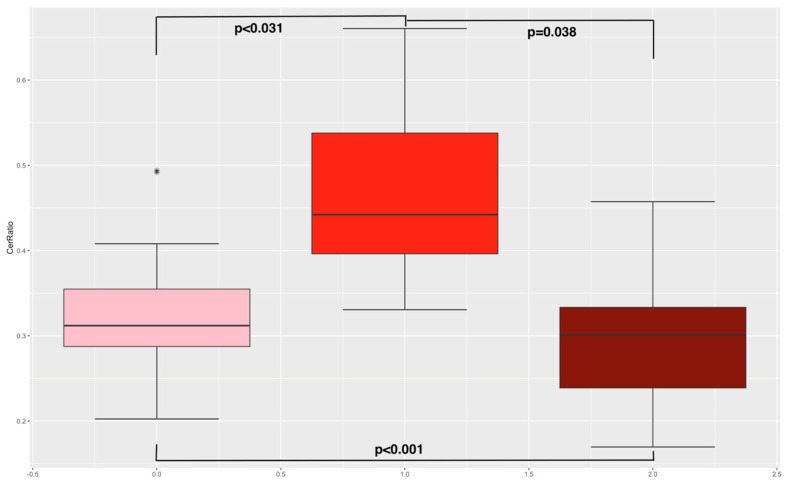
Ceramide ratio. This figure presents boxplots including the ceramide ratio distribution in the three study cohorts. The left boxplot indicates the RYGB cohort, the middle the obese, and the right the normal-weight cohort. The *p*-values for the unpaired *t*-test are also reported. The star indicates an outlier.

Next, an unpaired *t*-test between the different cohorts was conducted (see Supplementary Table S6). Therefore, lower ceramide risk scores in the RYGB cohort and the control group (normal weight) compared to the obese cohort could be determined (7.42 and 7.17 vs. 9.34, *p* = 0.025 and *p* = 0.026). No differences in ceramide risk scores between the RYGB cohort and the control group were found (7.42 vs. 7.17, *p* = 0.769). Lower levels of ceramide ratios Cer(d18:1/18:0)/Cer(d18:1/16:0) in the RYGB cohort compared to the obese cohort were analyzed (0.33 vs. 0.47, *p* < 0.001 (see Figure 2)). Further, lower levels of ceramide ratios Cer(d18:1/18:0)/Cer(d18:1/16:0) were found in the normal-weight cohort compared to the obese cohort (0.29 vs. 0.47, *p* < 0.001). No differences in ceramide ratios between the RYGB cohort and normal-weight cohort could be observed. As already published, pregnant women with a history of RYGB surgery were more insulin-sensitive (measured with SI Pacini: 0.69 vs. 2.27 (*p* = 0.002); calculated sensitivity index (CSI): 2.59 vs. 0.79 (*p* = 0.004); and HOMA index: 1.31 vs. 6.08, *p* = 0.022); had a higher disposition index, indicating stronger pancreatic beta cell function (DI Pacini: 355.16 vs. 151, *p* = 0.001); and had lower total body fat compared to the obese cohort (36.62% vs. 46.73%, *p* = 0.011) [7].

### 3.2. Correlation of Ceramide Risk Score/Ceramide Ratio and Glucose Metabolism in the RYGB Cohort

Looking at the correlation of ceramide risk scores on lipid and glucose parameters in the RYGB cohort, only a positive correlation of ceramide risk score and body fat was found (r = 0.650, *p* = 0.012 (see Table 1)).

In the RYGB cohort, the ceramide ratio was slightly positively correlated with the disposition index, indicating increased beta cell function in insulin resistance (r = 0.480, *p* = 0.044).

### 3.3. Correlation of Ceramide Risk Score/Ceramide RATIO and Glucose Metabolism in Individuals without Bariatric Surgery

Moreover, the correlation of the ceramide ratio on metabolic variables was investigated in individuals without prior bariatric surgery (see Table 2). Concerning the ceramide risk score, a negative correlation to the Matsuda index (r = −0.376, *p* = 0.031), sensitivity index (r = −0.364, *p* = 0.041), and calculated sensitivity index (r = −0.358, *p* = 0.044) in individuals without RYGB could be found. Further, a positive correlation of ceramide risk score with HbA1c levels (r = 0.384, *p* = 0.036) and body fat percentage (r = 0.602, *p* = 0.001) was investigated. Regarding the ceramide ratio, a negative correlation to the Matsuda index (r = −0.455, *p* = 0.008), sensitivity index (r = −0.622, *p* < 0.001), disposition index (r = −0.515, *p* = 0.003), calculated sensitivity index (r = −0.621, *p* < 0.001), and HDL cholesterol (r = −0.452, *p* = 0.009) was found. Next, a positive correlation of ceramide ratio and HOMA index (r = 0.519, *p* = 0.002), triglyceride levels (r = 0.492, *p* = 0.004), HbA1c levels (r = 0.637, *p* < 0.001), and body fat (r = 0.702, *p* < 0.001) was determined.

## 4. Discussion

In summary, our findings might indicate that lower ceramide risk scores and ceramide ratios in pregnancy could be related to lower cardiometabolic risk.

Normally, ceramides are used as the back bone of the cell membrane [2]. If there are excessive amounts of ceramides, they are also responsible for initiating cell death via intracellular signaling [2]. Nevertheless, the influence of ceramides depends on their added fatty acyl chains [14]. Past studies determined that higher levels of ceramide with long fatty acyl chains contribute to insulin resistance [28,34,35]. Previous studies determined a pathological increase in adipose tissue leading to disturbances in the control of the lipid storage, adipocyte turn-over, and hormone secretion in metabolic syndrome and type 2 diabetes mellitus [30]. Additionally, metabolites of ceramides, such as sphingosine, correlate with cardiovascular disease [27,36,37]. Further abnormalities in sphingolipid metabolism are related to adipose tissue-induced inflammation, which is mediated by cytokines such as tumor necrosis factor-α, interleukins, and C-reactive protein [27,38,39]. This inflammation counts as one of the crucial characteristics of obesity, diabetes mellitus, and cardiovascular disease risk [27,38,39]. In genetically modified mice, a study reported that through deletion of the enzyme dihydroceramide desaturase 1, by which the backbone of the ceramide receives a conserved double-bond, a decrease in insulin resistance could be investigated [28]. Also, our study demonstrated that high ceramide risk scores were associated with insulin resistance as measured by implemented indices in pregnant females without prior bariatric surgery. Further, the previously described ceramide ratio Cer(d18:1/18:0)/Cer(18:1/16:0), which is able to predict type 2 diabetes mellitus [24], is also associated with insulin resistance as measured by implemented indices in pregnant females without prior bariatric surgery in our study. A previous study was not able to prove that this ceramide ratio is a predictive value of gestational diabetes mellitus in pregnant females [25]. Still, concerning pregnant females with a history of RYGB, ceramide risk scores had no correlation to glucose metabolism, but the ceramide ratio was positively correlated with the disposition index, indicating increased beta cell function in insulin resistance. Previous literature reports a significant decrease in ceramides with long fatty acyl chains after RYGB. This decrease is most severe in the first 6 months after bariatric surgery [40]. Next, the level of these ceramides two years after bariatric surgery was able to predict diabetes mellitus risk in the following 12 years after RYGB [40]. In our study, the median time after RYGB is 4 years, which might affect the correlations of ceramide risk score and ceramide ratio on metabolic parameters. Still, in our study, the ceramide ratio had a positive correlation to the disposition index, indicating increased beta cell function in insulin resistance in pregnant females after RYGB. Nevertheless, it is known that individuals after RYGB have improved insulin secretion and might therefore also have higher disposition indices [41]. It might be postulated that more correlations of ceramide risk score and ceramide ratio on metabolic parameters would be investigated earlier after bariatric surgery. Further, this study investigated differences in ceramide risk scores, and, in particular, the obese cohort had higher concentrations compared to the RYGB and normal-weight cohort. Moreover, the obese cohort had lower insulin sensitivity indices and disposition indices, which indicates reduced beta cell function in insulin resistance compared to the normal-weight and RYGB cohort. However, the ceramide risk score and ceramide ratio had fewer correlations with metabolic indices in pregnant females after RYGB, suggesting that the lower cardiometabolic risk is not only caused by reduced ceramide concentrations.

Moreover, past studies investigated a lower frequency of gestational diabetes and better insulin sensitivity in pregnant females after RYGB compared to control subjects due to weight loss and hormonal changes including GLP-1 levels [10,11,27]. The fast improvement of insulin sensitivity in individuals after RYGB might be explained by the combination of alternations in gut peptide secretion and the missing of the foregut, as well as a decrease in inflammatory status caused by the massive decrease in visceral adipose tissue [27]. In obesity, an accumulation of fatty acids and triglyceride in non-adipose tissue, such as pancreatic islets, skeletal muscle, heart, and hepatocytes, takes place, which leads to cellular dysfunction [27]. This ectopic fat accumulation induces inflammation and therefore contributes to insulin resistance [27]. In our study, lower BMI levels were investigated in the RYGB cohort, which could also cause lower ceramide concentrations through the mechanism just mentioned.

Ceramide concentrations and ceramide risk scores are already used as a predictive biomarker for cardiovascular disease [42]. As is known, excessive deposits of lipids in organs aside from fat tissue, for example, in blood vessel walls and the heart, cause cardiovascular disease [2]. In healthy individuals, β-oxidation metabolizes free fatty acids into ATP [2]. Nevertheless, in the hypercaloric state, the excessive free fatty acids become triglycerides by being packed into a glycerol backbone [2]. These triglycerides can be stored in the cells as lipid droplets [2]. However, sometimes, this pathway becomes saturated and, therefore, lipids accumulate [2]. These lipids, including ceramides, start to disturb heart and vessel function and lead to cardiovascular disease [2]. Therefore, the finding of lower ceramide risk scores and ceramide ratios in the RYGB cohort compared to the obese study cohort could suggest that the RYGB might have a better cardiovascular risk profile compared to obese pregnant females. In our analyses, no difference in ceramide risk scores or ceramide ratios between females after RYGB and normal-weight females can be found. Further, the ceramide ratios were positively associated with triglyceride levels and negatively with HDL cholesterol in pregnant females without prior bariatric surgery. Nevertheless, no significant differences in triglyceride levels and cholesterol/HDL quotient between the obese and the RYGB cohort could be found. Previous studies have already determined that individuals have a lower 10-year and lifetime cardiovascular disease risk after RYGB compared to pre-surgery [5]. This is why bariatric surgery not only regulates weight but also improves long-term cardiovascular disease risk [5]. Moreover, participants with type 2 diabetes mellitus were randomized into two different intervention groups: one underwent bariatric surgery and one was treated with antidiabetic medication. It was investigated that after bariatric surgery individuals with prior type 2 diabetes mellitus had a lower 10-year risk of lethal and nonlethal coronary heart disease and stroke, as measured via the UKPDS risk equation, compared to obese type 2 diabetes mellitus individuals only treated with antidiabetic medications [43]. Further, individuals after bariatric surgery had lower frequencies of antidiabetic, antihypertensive, and lipid-lowering medications [43]. The suggested reason for these findings is the significant weight loss after bariatric surgery and hormonal changes, including GLP-1 levels [27,44]. Nevertheless, in our study, no correlation between gastric inhibitory polypeptide or glucagon-like peptide and ceramide levels could be seen. Still, GLP-1 levels are drastically postprandially increasing in RYGB individuals, leading to an increased insulin and C-peptide secretion [9]. Also, for glucagon, an elevation is reported up to 6 months after RYGB, and GIP is mostly decreased after RYGB in individuals with diabetes mellitus [45]. 

We have to report the following limitations. First, it might seem that this study has a low number of participants. However, the number of participants in this cross-sectional clinical study is comparable with the number in previously published work [46]. Second, the conclusions made in this study result from correlations and therefore not from causative relationships.

## 5. Conclusions

Nevertheless, to our knowledge, our study is the first one to measure ceramide risk scores and the ceramide ratio Cer(d18:1/18:0)/Cer(d18:1/16:0) in pregnant females after RYGB surgery and, further, to compare it to normal-weight and obese pregnant females. Therefore, our work could suggest that ceramide risk score and ceramide ratio might predict metabolic risk in pregnant females. Even in pregnant females after RYGB, the ceramide ratio level might suggest better cardiometabolic health to some extent. However further studies are required to understand the role of specific ceramides in pregnancy fully.

## Figures and Tables

**Table 1:** Correlation of ceramide risk score/ceramide ratios and metabolic parameters in the RYGB cohort.

Variable	Ceramide Risk Score Pearson Correlation (*p*-Value)	Ceramide Risk Score 95% Confidence Interval	Ceramide Ratio Pearson Correlation (*p*-Value)	Ceramide Ratio 95% Confidence Interval
Matsuda Index	−0.037 (0.883)	−0.50–0.44	−0.150 (0.553)	−0.58–0.34
HOMA-IR	−0.051 (0.842)	−0.51–0.43	0.126 (0.620)	−0.36–0.56
Sensitivity index (SI Pacini)	−0.243 (0.331)	−0.64–0.25	−0.347 (0.158)	−0.70–0.14
Disposition index (DI Pacini)	−0.122 (0.630)	−0.56–0.37	0.480 (0.044)	0.02–0.77
Calculated sensitivity index (CSI)	−0.199 (0.428)	−0.61- 0.29	−0.281 (0.259)	−0.66–0.21
Triglycerides mg/dL	0.269 (0.485)	−0.48–0.79	0.111 (0.776)	−0.60–0.72
Cholesterol total mg/dL	0.354 (0.350)	−0.41–0.82	0.019 (0.962)	−0.65–0.67
HDL cholesterol mg/dL	−0.091 (0.816)	−0.71–0.61	−0.543 (0.139)	−0.88–0.20
Chol/HDL Quotient	0.319 (0.402)	−0.44–0.81	0.415 (0.266)	−0.34–0.85
LDL cholesterol mg/dL	0.364 (0.336)	−0.40–0.83	0.259 (0.501)	−0.49–0.79
HbA1c (%)	0.295 (0.379)	−0.37–0.76	−0.010 (0.978)	−0.61–0.59
Body fat in percentage	0.650 (0.012)	0.18–0.88	0.345 (0.228)	−0.23–0.74
Ultra-sensitive CRP mg/dL	0.322 (0.335)	−0.34–0.77	−0.185 (0.586)	−0.71–0.47
Area under the Curve GIP	0.408 (0.092)	−0.07–0.74	0.420 (0.083)	−0.06–0.74
Area under the Curve GLP	−0.220 (0.380)	−0.62–0.28	−0.317 (0.200)	−0.68–0.18
Area under the curve Glucagon	−0.173 (0.493)	−0.59–0.32	−0.343 (0.164)	−0.70–0.15

This table presents the Pearson correlation of the ceramides risk scores/ceramide ratio Cer(d18:1/18:0)/Cer(d18:1/16:0) and the mentioned variables. LDL = low-density lipoprotein; HDL = high-density lipoprotein; GIP = gastric inhibitory polypeptide; GLP = glucagon-like-peptide.

**Table 2:** Correlation of ceramide risk score/ceramide ratios and metabolic parameters in individuals without bariatric surgery.

Variable	Ceramide Risk Score Pearson Correlation (*p*-Value)	Ceramide Risk Score 95% Confidence Interval	Ceramide Ratio Pearson Correlation (*p*-Value)	Ceramide Ratio 95% Confidence Interval
Matsuda Index	−0.376 (0.031)	−0.64–0.04	−0.455 (0.008)	−0.69–0.13
HOMA-IR	0.115 (0.526)	−0.24–0.44	0.519 (0.002)	0.21–0.73
Sensitivity index (SI Pacini)	−0.364 (0.041)	−0.63–0.02	−0.622 (<0.001)	−0.79–0.35
Disposition index (DI Pacini)	−0.114 (0.534)	−0.45–0.24	−0.515 (0.003)	−0.73–0.20
Calculated sensitivity index (CSI)	−0.358 (0.044)	−0.63–0.01	−0.621 (<0.001)	−0.80–0.35
Triglycerides mg/dL	0.347 (0.052)	−0.01–0.62	0.492 (0.004)	0.17–0.72
Cholesterol total mg/dL	0.062 (0.737)	−0.29–0.40	−0.301 (0.095)	−0.59–0.05
HDL cholesterol mg/dL	−0.199 (0.275)	−0.51–0.16	−0.452 (0.009)	−0.69–0.12
Chol/HDL Quotient	0.313 (0.081)	−0.04–0.60	0.243 (0.181)	−0.12–0.55
LDL cholesterol mg/dL	0.078 (0.671)	−0.28–0.42	−0.267 (0.139)	−0.56–0.09
HbA1c (%)	0.384 (0.036)	0.03–0.65	0.637 (<0.001)	0.36–0.81
Body fat in percentage	0.602 (0.001)	0.27–0.81	0.702 (<0.001)	0.43–0.86
Ultra-sensitive CRP mg/dL	0.271 (0.148)	−0.10–0.58	0.330 (0.075)	−0.03–0.62
Area under the Curve GIP	−0.066 (0.734)	−0.42–0.31	−0.324 (0.086)	−0.62–0.05
Area under the Curve GLP	−0.020 (0.912)	−0.37–0.33	−0.082 (0.655)	−0.42–0.27
Area under the curve Glucagon	0.084 (0.643)	−0.27–0.42	−0.059 (0.742)	−0.39–0.29

This table presents the Pearson correlation of the ceramides risk scores/ceramide ratio Cer(d18:1/18:0)/Cer(d18:1/16:0) and the mentioned variables. LDL = low-density lipoprotein; HDL = high-density lipoprotein; GIP = gastric inhibitory polypeptide; GLP = glucagon-like-peptide.

## Data Availability

The data presented in this study are available on request from the corresponding author due to ethical reasons.

## Supplementary Materials

The following supporting information can be downloaded at: https://www.mdpi.com/article/10.3390/nu16162704/s1, Table S1: Mass transitions for ceramides and their corresponding deuterated internal standards. Table S2: Within- and between-day precisions (expressed as RSD) and accuracies (expressed as bias) for ceramide d18:1/16:0. Table S3: Within- and between-day precisions (expressed as RSD) and accuracies (expressed as bias) for ceramide d18:1/18:0. Table S4: Within- and between-day precisions (expressed as RSD) and accuracies (expressed as bias) for ceramide d18:1/24:0. Table S5: Within- and between-day precisions (expressed as RSD) and accuracies (expressed as bias) for ceramide d18:1/24:1. Table S6: Baseline Characteristics.
