# Supplementary material for: Investigating a New Way to Assess Metabolic Risk in Pregnant Females with Prior RYGB Surgery
Source: Nutrients. 2024 Aug 14;16(16):2704. doi: 10.3390/nu16162704 (PMC11357170; doi:10.3390/nu16162704)
Supplement: Supplementary file 1 [file nutrients-16-02704-s001.zip › nutrients-3145164-supplementary.pdf]

Supplemental Materials

Determination of four Ceramides in EDTA plasma via LC-MS/MS

All samples were analyzed using liquid chromatography–tandem mass spectrometry with a 5500 QTrap system (Sciex, Framingham, MA, USA) equipped with a TurboIon Source for electrospray ionization. The chromatographic system consisted of a Thermo Scientific Ultimate 3000 (Waltham, MA, USA). Ceramides and corresponding deuterated standards were obtained from Avanti Polar Lipids: ceramide d18:1/16:0 (Avanti 860516P), ceramide d18:1/18:0 (Avanti 860518P), ceramide d18:1/24:0 (Avanti 860524P), ceramide d18:1/24:1 (Avanti 860525P), and the deuterated Ceramide Lipidomix Mass Spec Internal Standard (Avanti 330713X). The individual standards were dissolved and further diluted with butanol–methanol (1:1).

Formic acid, water, and 1-butanol were obtained from Merck, ammonium acetate for MS from Sigma-Aldrich, acetonitrile and 2-propanol from Supelco, fatty acid free Albumin fraction V from Roth, and PBS-Buffer from Lonza Bio.

For the analysis of plasma samples, a Luna Omega C18 (2.6 µm, 100A, 50 x 2.1 mm, Phenomenex, Torrance, CA, USA) equipped with a Security Guard Ultra Cartridge C18 was used at an oven temperature of 60°C. Mobile phase A consisted of 10 mM ammonium acetate in 0.1% aqueous formic acid (v/v). Mobile phase B consisted of 10 mM ammonium acetate in acetonitrile–2-propanol (4:3, v/v) with 0.1 % formic acid (v/) at a flow rate of 0.7 mL/min and a gradient elution program as follows: 80% B (held for 0.25 min) with a linear increase of up to 100% B over 1.25 min (held for 1 min) and back to 80% B within 1 min (held for 0.5 min).

The mass spectrometer was operated in the positive electrospray ionization mode. Quantification was performed via multiple reaction monitoring (MRM). Mass transitions are listed in Table S1.

Table S1: Mass transitions for ceramides and their corresponding deuterated internal standards.

component	precursor Ion (m/z)	product Ion (m/z) quantifier	product Ion (m/z) qualifier
Cer d18:1/16:0	538.3	264.2	520.4
d7-Cer d18:1/16:0	545.3	271.2	-
Cer d18:1/18:0	566.4	264.2	548.4
d7-Cer d18:1/18:0	573.4	271.2	-
Cer d18:1/24:0	650.5	264.2	632.5
d7-Cer d18:1/24:0	657.5	271.2	-
Cer d18:1/24:1	648.4	264.2	630.5
d7-Cer d18:1/24:1	655.4	271.2	-

A total of 5 % bovine albumin in PBS buffer was used for establishing six calibrators and three quality controls: ceramides d18:1/16:0 and d18:1/18:0 in the range of 0.01 to 2.0 nmol/mL and ceramides d18:1/24:0 and d18:1/24:1 in the range of 0.1 to 10.0 nmol/mL.

For sample preparation, 10 µL internal standard solution (2.0 nmol d7-Cer 16:0/d7-Cer 24:0 and 1.0 nmol d7-Cer 18:0/d7-Cer 24:1 in 1 mL methanol), 10µL water (to avoid precipitation), and 20 µL of plasma were transferred to Eppendorf cups, precipitated with 200 µL 1-butanol–methanol (1:1), vortexed for 5 seconds, and centrifuged for 5 min at 20.800 x g. The supernatant was transferred to vials with glass insert; 2 µL of sample solution was injected into the LC-system.

The method for the quantification of four ceramides in EDTA plasma was validated according to the EMA guideline (guideline on bioanalytical method validation, EMEA/CHMP/EWP/192217/2009 Rev. 1 Corr. 2**) and ICH guideline M10 on bioanalytical method validation (2019, draft).

Validation data

Selectivity (visibility of interferences) was tested in six different EDTA plasmas; no interferences were observed. Carry over (blank run after highest calibrator) was below the acceptance criteria of 20% for the analytes and below the acceptance criteria of 5% for the internal standards. LLOQ was determined as the lowest concentration of the calibration curve; results for precision and accuracy can be found in Table S1. The calibration curve was calculated with 1/x weighting and linear regression. Four calibration curves were analyzed during validation, showing correlation coefficients for ceramide d18:1/16:0 between r = 0.997 and 0.999, for ceramide d18:1/18:0 between 0.994 and 0.999, for ceramide d18:1/24:0 between 0.993, and 0.997 and for ceramide d18:1/24:1 between 0.992 and 0.997.
Within-day and between-day precision and accuracy for four QC levels in 5% BSA (LLOQ–QC, low QC, medium QC, and high QC) and three QC levels in a plasma pool are shown in Tables S1-4.

Table S2: Within- and between-day precisions (expressed as RSD) and accuracies (expressed as bias) for ceramide d18:1/16:0.

		**Within-day precision and accuracy (*n* = 5)**			**Between-day precision and accuracy (*n* = 9)**		
	**Nominal concentration (nmol/mL)**	**Mean concentration (nmol/mL)**	**RSD (%)**	**Bias (%)**	**Mean concentration (nmol/mL)**	**RSD (%)**	**Bias (%)**
QCs in 5% BSA	0.010	0.011	5.8	10.0	0.010	10.2	3.3
	0.030	0.322	11.9	7.3	0.030	5.8	1.1
	0.600	0.629	3.5	4.9	0.603	4.3	0.5
	1.600	1.596	2.4	-0.2	1.581	5.9	-1.2
QCs in plasma pool	0.243	0.255	3.8	-	0.254	4.8	-
	0.943	0.990	7.2	-	0.981	3.3	-
	1.543	1.572	2.2	-	1.659	8.9	-

Table S3: Within- and between-day precisions (expressed as RSD) and accuracies (expressed as bias) for ceramide d18:1/18:0.

		**Within-day precision and accuracy (n = 5)**			**Between-day precision and accuracy (n = 9)**		
	**Nominal concentration (nmol/mL)**	**Mean concentration (nmol/mL)**	**RSD (%)**	**Bias (%)**	**Mean concentration (nmol/mL)**	**RSD (%)**	**Bias (%)**
QCs in 5% BSA	0.010	0.010	11.9	-2.0	0.011	7.9	5.6
	0.030	0.031	4.3	4.7	0.031	12.6	4.8
	0.600	0.670	3.4	11.7	0.631	5.9	5.2
	1.600	1.772	3.4	10.8	1.641	6.3	2.6
QCs in plasma pool	0.090	0.103	3.7	-	0.103	4.0	-
	0.790	0.927	2.5	-	0.916	9.1	-
	1.390	1.594	5.3	-	1.642	5.3	-

Table S4: Within- and between-day precisions (expressed as RSD) and accuracies (expressed as bias) for ceramide d18:1/24:0.

		**Within-day precision and accuracy (n = 5)**			**Between-day precision and accuracy (n = 9)**		
	**Nominal concentration (nmol/mL)**	**Mean concentration (nmol/mL)**	**RSD (%)**	**Bias (%)**	**Mean concentration (nmol/mL)**	**RSD (%)**	**Bias (%)**
QCs in 5% BSA	0.100	0.094	4.8	-6.4	0.096	5.5	-4.4
	0.300	0.302	4.6	0.8	0.334	7.1	11.5
	4.000	4.154	5.9	3.9	4.160	4.7	4.0
	8.000	7.873	3.8	-1.6	7.840	4.5	-2.0
QCs in plasma pool	2.834	2.849	5.3	-	3.021	8.0	-
	5.834	6.253	2.6	-	6.433	9.5	-
	8.834	8.758	4.5	-	9.549	5.1	-

Table S5: Within- and between-day precisions (expressed as RSD) and accuracies (expressed as bias) for ceramide d18:1/24:1.

		**Within-day precision and accuracy (n = 5)**			**Between-day precision and accuracy (n = 9)**		
	**Nominal concentration (nmol/mL)**	**Mean concentration (nmol/mL)**	**RSD (%)**	**Bias (%)**	**Mean concentration (nmol/mL)**	**RSD (%)**	**Bias (%)**
QCs in 5% BSA	0.100	0.083	6.3	-16.8	0.094	7.2	-6.2
	0.300	0.300	4.6	-0.1	0.323	6.0	7.7
	4.000	4.332	1.7	8.3	4.123	5.6	3.1
	8.000	7.913	2.3	-1.1	7.774	5.1	-2.8
QCs in plasma pool	0.806	0.900	2.3	-	0.900	3.0	-
	3.806	4.315	0.7	-	4.231	5.8	-
	6.806	7.083	4.3	-	7.437	5.3	-

Baseline Characteristics

Table S6. Baseline characteristics.

**Variable**	**Obese females**	**Normal-weight females**	**Females after RYGB**	**ANOVA**
**Ceramide Risk Score**	9.34 ± 2.68 *	7.17 ± 2.92 **	7.42 ± 2.29	0.033
**Cer(d18:1/16:0) (pmol/mL)**	347±68 *	367±61	297±46 ***	<0.001
**Cer(d18:1/18:0) (pmol/mL)**	165±58 *	107±35 **	99±32	0.598
**Cer(d18:1/24:1) (pmol/mL)**	1388±319	1321±416	1201±270	0.294
**Cer(d18:1/24:0) (pmol/mL)**	2575±727 *	3127±692 **	2016±591 ***	<0.001
**Ceramide Ratio**	0.47±0.10 *	0.29±0.07 **	0.33±0.08	0.322
**BMI before pregnancy kg/m2**	37.94 ± 2.97 *	21.70 ± 2.10 **	28.44 ± 5.09 ***	<0.001
**Matsuda index**	2.28 ± 1.30 *	8.95 ± 7.87 **	4.59 ± 2.28 ***	0.239
**HOMA-IR**	6.08 ± 6.28 *	1.37 ± 0.71 **	1.31 ± 0.64	<0.001
**Sensitivity index (SI Pacini)**	0.69 ± 0.35 *	3.49 ± 1.68 **	2.27 ± 1.07	<0.001
**Disposition index (DI Pacini)**	151 ± 81.08 *	661.05 ± 302.79 **	355.16 ± 176.40 ***	<0.001
**Calculated sensitivity index (CSI)**	0.79 ± 0.40 *	3.82 ± 1.86 **	2.59 ± 1.30	<0.001
**HbA1c (%)**	5.15 ± 0,46	4.68 ± 0.36	4.66 ± 0.43	0.023
**Body fat in percentage**	46.73 ± 3.00 *	34.75 ± 3.00 **	36.62 ± 6.27	<0.001
**Time after RYGB**	-	-	Median: 4 years<br>Minimum: 1 year<br>Maximum: 12 years	
**Weight loss after RYGB**	-	-	Median: 47kg<br>Minimum: 39kg<br>Maximum: 97kg	

[1] This table reports the mean value and standard deviation for the used variables divided into obese females, the control group (normal-weight females), and the study group (females after RYGB). Further, it reports the *p*-values of the ANOVA analyses. * indicates that the *p*-value of the unpaired t-tests between obese females and females after RYBG are significant ($p<0.05$); ** indicates that the *p*-value of the unpaired t-tests between obese females and normal-weight females are significant; *** indicates that the *p*-value of the unpaired t-tests between normal-weight females and females after RYBG are significant.
